# Progress and Challenges of Immunotherapy Predictive Biomarkers for Triple Negative Breast Cancer in the Era of Single-Cell Multi-Omics

**DOI:** 10.3390/life13051189

**Published:** 2023-05-16

**Authors:** Jiangnan Yu, Zhikun Guo, Lei Wang

**Affiliations:** International Cancer Center, Shenzhen University Medical School, Shenzhen 518054, China; jiangnanyu@szu.edu.cn (J.Y.); 2100243054@email.szu.edu.cn (Z.G.)

**Keywords:** TNBC, predictive biomarker, immunotherapy, multi-omics

## Abstract

Triple-negative breast cancer (TNBC) is a highly aggressive subtype of breast cancer with a poor prognosis. Despite conventional treatments, including surgery, radiation, and chemotherapy, the overall response rate to PD-1/PD-L1 immune checkpoint inhibitors remains low, with limited predictive significance from current biomarkers such as PD-L1 expression, tumor-infiltrating lymphocytes (TILs), and tumor mutational burden (TMB). To address this challenge, recent advancements in single-cell sequencing techniques have enabled deeper exploration of the highly complex and heterogeneous TNBC tumor microenvironment at the single-cell level, revealing promising TNBC predictive biomarkers for immune checkpoint inhibitors. In this review, we discuss the background, motivation, methodology, results, findings, and conclusion of multi-omics analyses that have led to the identification of these emerging biomarkers. Our review suggests that single-cell multi-omics analysis holds great promise for the identification of more effective biomarkers and personalized treatment strategies for TNBC patients.

## 1. Introduction

Breast cancer is a frequent malignant disease in women worldwide and is categorized into three major subtypes based on the molecular level: human epidermal growth factor receptor 2 (HER2), estrogen receptor (ER), and progesterone receptor (PR) [1]. TNBC is clinically negative for expression of the HER2, ER, and PR, which is more likely to recur than the other two subtypes (absence of HER2 or absence of ER and PR) [2]. TNBC is often characterized by a high histological grade, strong invasiveness, and high rate of metastasis. TNBC has a poor prognosis due to its aggressive clinical characteristics and lack of response to receptor-targeted therapy [3]. 

Therefore, there is an urgent need for more effective treatment for TNBC. TNBC accounts for 20% of breast cancer [4]. In recent years, remarkable progress has been made in exploiting the intrinsic mechanism of the host immune system to eliminate cancer cells. The advancement in immunotherapy provides a potential novel therapeutic approach for managing this devastating subtype of breast cancer. It is anticipated that immunotherapy intervention will elicit a specific response that targets and eradicates tumor cells while preserving normal cells. Diverse immunotherapy techniques have been developed and investigated, including the use of neutralizing or inhibitory antibodies to block immune checkpoints, induction of cytotoxic T lymphocytes (CTLs), adoptive cell transfer-based therapy, and modulation of the tumor microenvironment to enhance CTL activity [4].

**Figure 1:**
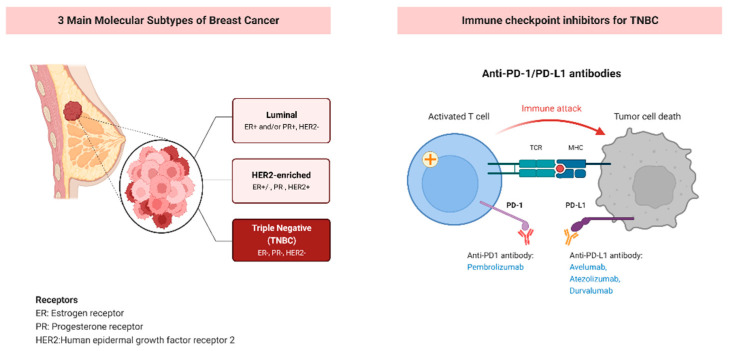
Immune checkpoint inhibitors for patients with TNBC.

TNBC patients may be given neoadjuvant treatment (chemotherapy before resection) in early stage tumors, which could shrink tumor size and protect normal breast tissue [5]. Immunotherapies also appear to be durable in metastatic TNBC, which suggests that immunotherapies may bring better treatment strategies to responding patients. Immune checkpoint antagonists targeting cytotoxic T lymphocyte-associated antigen-4 (CTLA-4), programmed cell death-1 (PD-1) and programmed death ligand-1 (PD-L1) have completely changed cancer treatment, induced lasting objective reactions, and sometimes translated into overall survival (OS) benefits of multiple cancer types including breast cancer [6] (Figure 1).

This review contributes to the recent exploration of the highly complex and heterogeneous TNBC tumor microenvironment at the single-cell level. We summarize the major contributions of single-cell multi-omics in TNBC research, including the identification of novel immune cell subpopulations and cellular interactions, the characterization of dynamic changes in tumor heterogeneity and clonal evolution during treatment, and the discovery of potential therapeutic targets and biomarkers for immune checkpoint inhibitors.

## 2. PD-1/PD-L1 Checkpoint Inhibitor in Triple Negative Breast Cancer

PD-1 and PD-L1 are important immunotherapy targets in TNBC treatment. PD-1 receptors are upregulated on activated T cells and bind to the related ligand, PD-L1. Through the interaction with PD-L1 on the surface of tumor cells and immune cells, the PD-1 signal antagonizes T cell activation during the immune response stage [7]. Some immune checkpoint inhibitors (ICIs) targeting PD-1 and PD-L1 have shown favorable treatment effects in TNBC patients.

Pembrolizumab—Humanized monoclonal antibodies that target PD-1 (pembrolizumab) improve event-free survival (EFS) in TNBC [8]. Patients with stage II-III TNBC usually receive neoadjuvant chemotherapy before surgery. Nevertheless, about 30% of patients will experience disease progression within five years after typical treatment, which indicates the need for more effective upfront treatment in TNBC [9]. Currently, data from the phase III KeYNOTe-522 trial shows that in this case, neoadjuvant pembrolizumab plus chemotherapy followed by adjuvant pembrolizumab has advantages over neoadjuvant chemotherapy (NCT03036488) [10]. Pembrolizumab has been tested in several clinical trials, demonstrating its safety and clinical activity across a range of tumor types [11,12]. These data led to FDA approval of pembrolizumab in combination with chemotherapy as of July 2021.

Avelumab, atezolizumab, durvalumab—ICIs, monoclonal antibodies against PD-L1 (avelumab, atezolizumab, durvalumab), have generated durable responses across many tumor types including TNBC [13]. Although avelumab and atezolizumab are already applied to ICI monotherapy in metastatic triple-negative breast cancer (mTNBC), low response rates have been observed in pretreated metastatic disease: in the phase Ib JAVELIN trail (NCT01772004), the overall response rate (ORR) of avelumab in 58 heavily pretreated patients was 5.2% [14], while the phase I trial of atezolizumab (NCTO1375842) resulted in an ORR of 10% in 115 pre-treatment patients, with no response observed in the PD-L1 negative subgroup [15]. The GeparNuevo trial (NCT02685059) demonstrated that durvalumab improved pathologic complete response (pCR) rates when durvalumab was started two weeks before chemotherapy, which was a subgroup analysis underpowered for significance testing [16].

## 3. Current Predictive Biomarkers for PD1/PD-L1 Checkpoint Inhibitors

In patients with advanced-stage TNBC, monotherapy with PD-1 or PD-L1 antibodies has limited efficacy and might only benefit a small portion of patients [17]; chemo-immunotherapy approaches have improved tumor progression-free and overall survival, but these trials have yet to undergo detailed biomarker analysis [5]. Overall, immunotherapy still faces some difficulties: therapeutic resistance, unclear mechanisms, and poor response (<20%), which indicates that more efficient biomarkers are needed to identify TNBC patients who can benefit from immunotherapies in prediction and prognosis.

Because of the low response rate of immunotherapy, established and developing prognostic and predictive biomarkers are important to clinical therapies guide. Some known biomarkers of breast cancer, such as PDL1, TILs, and TMB, are helpful in the management of breast cancer.

### 3.1. Intratumoral PD-L1 Expression and Tumor Infiltrating Lymphocytes

The assessment of TILs and tumor PD-L1 expression has been proposed as potential predictors of clinical outcome in breast cancer. However, the reliability of these biomarkers in predicting the response to immunotherapy in early stage TNBC remains uncertain, as response to checkpoint inhibitors has been observed in tumors lacking PD-L1 expression [18]. There were 20% percent tumor cells that are PD-L1 positive in TNBC, and PD-L1 present in 20% of TNBC samples [19]. The inhibitory interactions between tumor-infiltrating immune cells and PD-1^+^ T cells associated with poor prognostic features [19]. PD-L1 can be measured and quantified on tumor or immune cells. Tumor PD-L1 negative patients can also benefit from ICIS because other immune cells can express PD-L1, and ICIs are activated the whole immune system. Nevertheless, recent clinical trials, such as KEYNOTE-119, have demonstrated that PD-L1 positivity alone may not be a sufficient biomarker to select patients who will benefit from pembrolizumab monotherapy in the metastatic setting [20,21].

In addition, TILs have been shown to be promising microenvironment biomarkers with independent predictive value for the clinical benefits of ICI. In the metastatic setting, CD8^+^ T cell infiltration, in particular, has been predictive of overall survival benefit with atezolizumab in IMpassion130 [22]. TILs seem to be slightly associated with PD-L1, but it has independent predictive value for the clinical benefits of ICI [23,24]. In early TNBC, an increase in TILs has been associated with improved disease-free survival, overall survival, and pathological complete response rate following neoadjuvant chemotherapy [25,26]. In metastatic TNBC, higher TIL levels have also been associated with improved prognosis. Despite its potential as a low-cost biomarker with additive predictive value to PD-L1 expression, no TILs test has yet entered routine clinical practice [21,22]. However, no TILs test has entered routine clinical practice, future research should further explore the potential of TILs as a predictive biomarker in ICI therapy, particularly in combination with PD-L1 expression.

### 3.2. Tumor Mutational Burden

Tumor mutational burden (TMB) is a metric used to measure the number of somatic mutations per megabase (mut/Mb) of DNA, typically determined through whole exome or gene panel sequencing. A recent analysis by Isaacs et al. found that breast cancer has a relatively low TMB of 2.63 muts/mb, with only 5% of tumors classified as hypermutated (>10 mut/MB) [20]. Breast cancer tumors with high TMB appear to be more sensitive to checkpoint inhibitors; However, there was no difference in OS among patients with high TMB breast cancer who received immunotherapy [27]. In 2020, the FDA approved pembrolizumab for the treatment of high TMB (≥10 mut/Mb) non-resectable or metastatic solid tumors that have progressed after previous treatment or have no alternative treatment options, making it a potential treatment option for patients with high TMB TNBC [18].

## 4. Predictive Biomarkers Revealed by Single-Cell Multi-Omics

### 4.1. T Cell Expansion and Differentiation

Although ICIs combined with neoadjuvant chemotherapy improves pCR and EFS in TNBC [28], only a subset of tumors responds to neoadjuvant ICI. To understand the response of which underlying mechanisms and associated markers determine neoadjuvant ICI treatment response, Bassez and Vos et al. conducted a single-cell multi-omics analysis of pre-treatment and on-treatment biopsies from treatment-naive patients receiving anti-PD1 (*n* = 29) or neoadjuvant chemotherapy before anti-PD1 (*n* = 11) therapy [29]. They found that one third of the tumors contained PD1-expressing T cells that clonally expanded after anti-PD1 therapy, regardless of tumor subtype, while some gene sets were positively or negatively correlated with T cell expansion following anti-PD1 treatment [29]. Clonal expansion of T cells underlies response to ICI therapy for several cancer types, such as melanoma or lung cancer, and the single-cell characterization of pre- and on-treatment biopsies of breast cancer are absent in the previous research [30,31,32].

By contrasting patients’ immune microenvironment alterations with and without emerging clonal expansion before and after treatment utilizing single-cell multi-omics, Bassez and Vos et al. revealed the regulation of differentiation of multiple immune cells in response to immunotherapy, and the possible mechanisms: the CD4^+^ and CD8^+^ T cell subtypes are the main targeted cells of anti-PD-1 therapy; the extent of differentiation and clonal proliferation of the lineage corresponding to CD8^+^ experienced T cells (CD8^+^_TEX_), type-1 helper (T_H1_) and follicular helper (T_FH_) cells can be used to predict response to anti-PD-1 therapy, and it is likely that anti-PD-1 therapy will further enhance the differentiation of these cells [29].

Furthermore, PD-L1-expressing macrophages such as CCR2^+^ or MMP9^+^ and multiple dendritic cell subtypes were positively associated with T cell expansion and treatment response [33,34], whereas the proportion of CX3CR1^+^ macrophages was negatively associated with clonal proliferation of T cells [35,36,37]. This study further found that the predominant cell type expressing PD-L1 in breast cancer is not tumor cells but macrophages and dendritic cells, whereas high expression of PD-L1 on macrophages and dendritic cells was predictive of immunotherapy response. In addition, macrophage phenotypes expressing PD-L1, including CCR2^+^ and MMP9^+^ macrophages, correlated positively with T cell expansion, which shows ICIs response. Inhibitory macrophages (CX3CR1^+^, C3^+^) were inversely correlated with T cell expansion, which shows limited ICIs response [29].

Therefore, T_EX_ cell abundance, T cell clonality, and richness were regarded as highly predictive markers of T cell expansion. Immune checkpoint markers or CD4^+^ T cell activation gene markers are also highly predictive, whether in the initial BC treatment or after neoadjuvant chemotherapy, anti-PD1 is given [29]. Interestingly, the expression of these markers in TNBC is more obvious than that in ER^+^ BC, which may explain why ICI has provided the greatest benefit in TNBC treatment so far [29].

Virassamy et al. revealed that tumor CD8^+^ T cells with tissue-resident memory phenotypes mediate local immunity and immune checkpoint reaction of breast cancer. This study explores the role of tissue-resident memory T (TRM) cells in breast cancer and their contribution to anti-tumor immunity and immune checkpoint blockade efficacy. The study found that intratumoral CD8^+^ T cells with a TRM-like phenotype display significantly enhanced cytotoxic capacity and provide local immune protection against tumor rechallenge. Treatment with anti-PD-1 and anti-CTLA-4 therapy resulted in the expansion of these intratumoral populations [38]. The study established two intratumoral sub-populations: one more enriched in markers of terminal exhaustion (TEX-like) and the other with a bona fide resident phenotype (TRM-like) [38]. A TRM gene signature extracted from tumor-free tissue was significantly associated with improved clinical outcomes in TNBC patients treated with checkpoint inhibitors. 

First, the team assumed that immune checkpoint inhibitor therapy targeted local tumor microenvironment, and CD8^+^ TRM cells might be crucial to its therapeutic effect on cancer; The phenotypic characteristics and cytokine requirements for the production and maintenance of different intratumoral CD8^+^ T cell subsets in cancer were established. They further confirmed the molecular differences of these intratumoral populations and proved that the CD69^+^CD103^+^ subgroup showed an increased expression of TEX-related genes (such as Tox and Eomes) [39] and showed a transcriptional spectrum similar to that of terminally depleted T cells in the context of chronic lymphocytic choriomeningitis virus (LCMV) [40]. The CD69^+^CD103^+^ subgroup showed enhanced anti-tumor function in mediating tumor lysis [38]. These T cell subsets were further verified by unbiased clustering of CD8^+^ single cell transcriptome data analyzed before ICB treatment. The two clusters distinguished by the expression of Itgae and Tox have significant transcriptional similarity with the large number of RNA sequences of the classified CD69^+^CD103^+^ and CD103^−^ subgroups, respectively.

Phenotypic and transcriptional studies have established two intratumoral subpopulations: one is richer in terminal failure markers (TEX-like), and the other has a real resident phenotype (tissue resident memory T (TRM-like)) [38]. The treatment of anti-PD-1 and anti-CTLA-4 led to the expansion of these tumor populations, and the TRM-like subgroup showed significantly enhanced cytotoxicity. TRM-like CD8^+^ T cells can also provide local immune protection against tumor challenge, and TRM gene markers extracted from tumor-free tissues are significantly related to the improvement of clinical prognosis of TNBC patients treated with checkpoint inhibitors [38].

It is reported that CD8^+^ tumor-infiltrating lymphocytes with TRM cell phenotypes are related to the good prognosis of TNBC patients. However, the relative contribution of CD8^+^ TRM cells to breast cancer anti-tumor immunity and immune checkpoint blocking efficacy is still unknown [38]. Overall, the study highlights the importance of TRM-like CD8^+^ T cells in breast cancer anti-tumor responses and ongoing protective immunity.

### 4.2. CXCL13^+^ CD4^+^ and CD8^+^ T Cells

In TNBC, the combining chemotherapy paclitaxel with PD-L1 checkpoint inhibitors atezolizumab did not benefit all patients [41,42]; to illuminate the different immune responses in TNBC patients (22 patients with TNBC pre- and on-treated with paclitaxel or its combination with atezolizumab), Zhang et al. leveraged single-cell multi-omics to investigate the dynamic map of tumor microenvironment and immune cells derived from peripheral blood. The tumor tissue and peripheral blood immune cells from patients with TNBC who received two treatment schemes were analyzed at the single cell level, and the tumor microenvironment and peripheral blood immune characteristics of response patients and non-response patients were compared. The dynamic changes of immune cells under different treatment strategies and the mechanism of anti-PD-L1 immunotherapy combined with paclitaxel chemotherapy in TNBC have been revealed as well as the selection of biomarkers. Zhang et al. found that the tumor microenvironment in response to patients was enriched with two groups of T cells with high expression of CXCL13 (CD8-CXCL13 and CD4-CXCL13) [43], and also highly expressed T cytotoxicity and exhaustion-related genes [44].

To investigate the connection systematically between the composition and proportion changes of different immune cells and the treatment effect, the research developed two indices: predictive index (Pi) and therapeutic index (Ti) [43]. Through the analysis of Pi and Ti, the researchers found that CD8-CXCL13 and CD4-CXCL13 at higher baseline levels can predict better immune treatment response, in addition, the proportion of these two groups of CXCL13^+^ T cells in response patients increased significantly after combined treatment [43]. 

In addition, researchers found that two groups of pro-inflammatory macrophages with high expression of CXCL9 and CXCL10 were enriched in the tumor microenvironment of response patients, and there was a significant positive correlation between these two groups of pro-inflammatory macrophages and CXCL13^+^ T cells [45,46]. CXCL9 and CXCL10 can participate in the recruitment of T cells [45], and the characteristic genes of proinflammatory macrophages are regulated by IFNG and TNF signals, indicating that there is a positive feedback signal between CXCL13^+^ T cells that play a killing function and proinflammatory macrophages that express CXCL9 and CXCL10 [43]. On the contrary, CXCL13^+^ T cells were hardly detected in the tumor microenvironment of non-responsive patients, but a large number of macrophages with immunosuppressive function were enriched [43]. It is noteworthy that the researchers found that the peripheral blood mononuclear cells of response patients showed pro-inflammatory characteristics, while the peripheral blood mononuclear cells of non-response patients showed anti-inflammatory characteristics, suggesting that the peripheral blood can reflect the tumor microenvironment characteristics to a certain extent [43].

### 4.3. Tumor-Resilient T Cell Assessed by Tres Model

In recent years, cancer immunotherapy represented by anti-PD-1/PD-L1, anti-CTLA4, and CAR-T has made considerable progress [47]. However, the effect of various immunotherapies on solid tumors is not satisfactory [47,48]. For T cells, a solid tumor is a battlefield with a suppressive environment [49]. The tumor will establish a microenvironment full of various immunosuppressive factors to suppress and differentiate the invading T-cell soldiers [49,50]. Despite being armed with various anti-cancer mechanisms, most T cells cannot persist in such a harsh environment [50].

They have developed a computational model called Tres (tumor-resilient T cell, https://resilience.ccr.cancer.gov/ accessed on 12 March 2023) by analyzing single-cell T-cell transcriptomes from ICI-treated melanoma or lung tumors to find the characteristics of T cells that are still active under the suppression of various inhibitors in solid tumors and predict the efficiency of T cells in immunotherapy [51,52,53]. Tres also identifies FIBP as a new checkpoint for T-cell immunometabolism and a possible new target for immunotherapy [54].

Tres is a computational model that uses single-cell transcriptome data to identify the characteristics of T cells resistant to immunosuppressive types, and Tres was also trained by TNBC-published single-cell transcriptomic profiles of T cells from responders and non-responders to ICIs [38]. It presented better predictive signature correlates in responders than non-responders in pre- and post-treatment of ICIs [49]. The application of single-cell sequencing technology in tumor research has produced a large number of single-cell gene expression profiles, depicting various states of T-cell subsets from tumors [53]. Tres, a computational model assesses the cytokines perceived by each T cell in the tumor environment; for example, TGFβ and PGE2 are common immunosuppressive factors [55], and TRAIL is the trigger of T cell in cell death [50]. If the downstream pathway of these cytokines is activated, it indicates that the T cell is in an unfavorable environment. At the same time, the health of T cells can be measured by the cell cycle and the expression of DNA replication pathway genes [55]. The activity of these pathways in suppressed or dying cells is often low. Based on the variables calculated and evaluated above, Tres looks for which T cells are under the pressure of various inhibitors, still remain healthy. These T cells are defined as tumor-resilient T cells (Tres) [54]. These Tres features demonstrate important clinical applications.

Based on the simplest correlation coefficient calculation, if these T cell samples are positively correlated with the characteristics of the tumor-resilient T cells model, the corresponding immunotherapy will achieve good results. If there is a negative correlation, the corresponding immunotherapy effect will be unfavorable [54]. It is particularly pointed out that the Tres model is almost correct in predicting the accuracy of patients with poor efficacy in cell therapy using only pre-manufacturing samples [54]. 

Zhang et al. analyzed the genetic characteristics of Tres. In 168 tumors and single-cell expression data from 19 kinds of cancer, the high expression of the FIBP gene in T cells almost indicates the low match of the Tres model, which means that T cells with high expression of the FIBP gene are not regarded as a tumor-resilient T cell [54]. Many solid tumors have high cholesterol concentrations [56]. Although an appropriate amount of cholesterol will guarantee the activity of T cells, excessive cholesterol concentration will greatly reduce the tumor-killing ability of T cells and lead to T cell exhaustion [56]. 

Therefore, the Tres model uses single-cell data to identify immunotherapy response biomarkers and predict cell therapy response from pre-manufacture samples, which provide an important research and development tool for cancer immunotherapy guidelines.

### 4.4. CD8^+^ T Cell-Intrinsic IL-6

Although immune checkpoint inhibitors (ICI) have been identified as effective cancer therapies, overcoming drug resistance remains a key challenge. Huseni et al. determined that interleukin 6 (IL-6) is associated with poor reaction to atezolizumab (anti-PD-L1) in large clinical trials of advanced renal cancer, breast cancer, and bladder cancer [57]. The pleiotropic cytokine IL-6 is associated with tumor progression and is supposed to affect anti-tumor immunity through a variety of mechanisms [58,59,60]. Plasma IL-6 has a negative effect on the survival rate of melanoma patients treated with ICI [61,62], and IL-6 appears to be a potential driver of ICI resistance [63,64,65]. 

In this study, Huseni et al. found that high levels of IL-6 are a characteristic of atezolizumab-resistant disease in patients with advanced cancer [57]. IL-6 inhibits the effector differentiation of CD8^+^ T cells (also known as cytotoxic T lymphocytes or CTLs), and high plasma IL-6 is associated with lower expression of effector genes in CTLs of cancer patients [57]. IL-6 impairs anti-PD-L1 efficacy by restricting the anti-tumor functions of cytotoxic T cells and IL-6-STAT3 signaling inhibits classical cytotoxic differentiation of CTLs in vitro [57]. In preclinical tumor models, blocking IL6R or gene ablation of intrinsic IL-6 signaling in CTLs, in combination with anti-PD-L1 therapy, enhances the anti-tumor CTL response, and improves tumor control [57].

In the PCD4989g clinical trial, patients with mTNBC treated with atezolizumab13, or in the IMvigor210 and IMvigor211 trials [66,67,68], patients with metastatic urothelial bladder cancer (UC), compared with the healthy control group, had elevated plasma IL-6, and was associated with low OS in multivariate survival analysis. According to the single-cell RNA sequencing, the circulating CTL of cancer patients with high plasma IL-6 levels showed a suppressed functional feature, and IL-6-STAT3 signal transduction inhibited the classical cytotoxic differentiation of CTL in vitro [57]. Therefore, based on clinical and experimental evidence, drugs targeting the IL-6 signal are reasonable partners for cancer patients and ICIs in combination treatment [57].

### 4.5. TME Phenotypes Do Not Respond to Checkpoint Inhibitors

In an effort to understand the lack of response to immune checkpoint inhibitors (ICI), Hammerl et al. conducted a study analyzing 681 triple-negative breast cancers (TNBCs) for spatial immune cell contextures in relation to clinical outcomes and pathways of T cell evasion [69,70,71]. Through this analysis, the authors identified three main spatial phenotypes: inflamed, excluded, and ignored, and recognized their association with clinical outcomes in TNBC and other cancer types [67]. The inflamed phenotype, characterized by the presence of intratumoral lymphocytes, is related to anti-PD-1 response, while the excluded and ignored phenotypes, characterized by lymphocytes restricted to the invasive margin or a lack of lymphocytes, respectively, are related to anti-PD-1 resistance [67].

Combined with multiple immunofluorescence and sequencing technology, Hammerl et al. revealed: immune excluded phenotypes (related to anti-PD-1 resistance), showed collagen-10 deposition, enhanced glycolysis, and TGFβ/VEGF pathway activation; immune ignored phenotypes (related to anti-PD1 resistance), showing high-density CD163^+^ myeloid cells or activating WNT/PPARγ pathways; inflamed phenotype, which was associated with anti-PD-1 response, exhibited necrosis, high-density CLEC9A^+^ dendritic cells, high TCR clonality, and enhanced expression of T cell co-inhibitory receptors [72]. These results suggest that the spatial immunophenotypes of primary TNBC have unique immune-determinants, as well as tumor microenvironment (TME) and immune response-mediated T cell escape pathway.

The TONIC test found that the proportion of inflammatory phenotype increased after cisplatin and doxorubicin induction treatment, which indicated that the spatial phenotype was plastic [73], while the cold TNBC (i.e., excluded and ignored) phenotypes can be remodeled, suggesting the possibility of treatment benefit for these two types of patients. Therefore, the immune excluded type and the ignored immunophenotypes in TNBC and metastatic TNBC validated by the gene classifier accurately do not respond to anti-PD1 treatment, which can be considered as a variant of cold tumor [72]. The spatial phenotype classifier demonstrated good predictive value, which could potentially improve the efficacy of anti-PD-1 treatment.

## 5. Conclusions

In the era of single-cell multi-omics, there has been significant progress and challenges in predicting immunotherapy biomarkers for triple-negative breast cancer. The use of single-cell multi-omics techniques has enabled the identification of novel biomarkers and molecular pathways that play a critical role in the response to immunotherapy. Additionally, these techniques have provided a deeper understanding of the complex immune landscape of triple-negative breast cancer and the heterogeneity of individual tumor cells, which has helped to refine biomarker discovery and validation. However, there are still many challenges that need to be addressed and other kinds of biomarkers are needed. One challenge is the lack of standardization in data analysis and interpretation across different studies, which can lead to inconsistencies in biomarker identification and validation. Another challenge is the limited sample size and heterogeneity of patient cohorts, which can affect the accuracy and reproducibility of biomarker discovery.

Furthermore, the integration of different multi-omics datasets is still in its early stages and requires more advanced computational methods and analytical tools. Despite these challenges, the use of single-cell multi-omics techniques offers great potential for identifying predictive biomarkers for immunotherapy in triple-negative breast cancer and for developing more personalized and effective treatment strategies.

Immunotherapies show the prospect of breast cancer treatment and the potential of activating the immune system to eliminate cancer cells. Inhibitors of PD-1/PD-L1 checkpoints can induce a long-lasting clinical response in some breast cancer patients with metastatic TNBC. Although some TNBC patients show PD-L1 negative expression in tumors, they can still benefit from ICIs. Intratumoral PD-L1 expression is highly heterogenous and PD-L1 expression on either cancer cells or immune cells can be changing dynamically. More importantly, we believe that the clinical efficacy of ICIs treatment requires stimulating the systemic anti-tumor immunity of TNBC patients and it is now reported that tumor-specific T cell activation in the tumor-draining lymph node can be targeted by ICI treatment [74].

**Figure 2:**
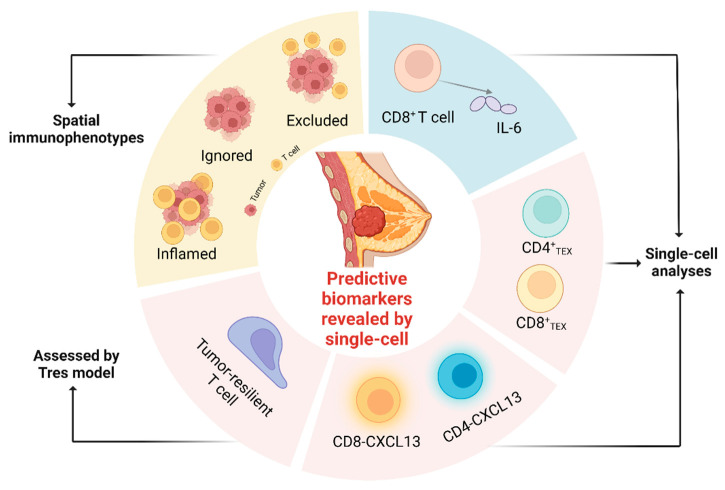
Summary of emerging anti-PD1/PD-L1 predictive biomarker revealed by scRNA multi-omics. Figures created with BioRender.com.

In addition, the combination of checkpoint inhibitors and chemo/targeted therapies in neoadjuvant first-line treatments has already demonstrated clinical benefit and potential. However, the major challenge is that the current biomarkers, such as intratumoral PD-L1 expression, TILs, and TMB, have limited predictability and reliability to select patients with TNBC. Therefore, there is an urgent need to develop novel predictive biomarkers by using deep multi-omics analysis at single-cell level. The involvement of multi-omics technology in TNBC predictive biomarker research has made promising discoveries so far (summarized in Figure 2). Future research, especially deep immune profiling of paired tumor, lymph node, and blood samples of pre- vs. post treatment are needed to further explore biomarkers during the ICI-induced systemic immune changes. 

## Data Availability

Not applicable.
